# Pure Air

**Published:** 1873-06

**Authors:** 


### PURE AIR.

Carbonic acid gas is produced by burning charcoal. If the door is closed this gas is increased in quantity every moment, until the room is so full of it that the healthiest man will die on the spot.

Every breath of air coming from the lungs is largely composed of carbonic acid gas, and if a room is crowded and shut up so close that no fresh air can get in, every person in the room will die in a few hours; its presence is very perceptible on going from out-doors in the morning into a room in which several persons have slept.

To prove that there is carbonic acid gas in the breath coming out of the lungs, it is only necessary to blow into half-a-glass of lime-water through a quill or straw. The water begins to turn white in a moment, by the carbon of the gas uniting with the lime in the water, making a solid substance, called the carbonate of lime.

Take a half-pint bottle, fill it over half full with lime-water, cork it, shake it well; if there is any of this poisonous gas in the air a white sediment will settle at the bottom—the more injurious the air, the more abundant will be the sediment. If there is much of this gas in the air a man is breathing, he will die at once, and as certainly as by a stroke of lightning. If he breathes a less amount he will die as certainly, but not so soon.

